# Temporal trends and patterns in suicidal ideation among adolescents in 23 countries from 2003 to 2021

**DOI:** 10.1038/s41598-025-19158-5

**Published:** 2025-10-08

**Authors:** Wonwoo Jang, Yejun Son, Jae E. Lee, Hyejun Kim, Seohyun Hong, Yeona Jo, Hanseul Cho, Hayeon Lee, Ho Geol Woo, André Hajek, Dong Keon Yon, Lee Smith

**Affiliations:** 1 Center for Digital Health, Medical Science Research Institute, Kyung Hee University College of Medicine, Seoul, South Korea; 2 Department of Medicine, Kyung Hee University College of Medicine, Seoul, South Korea; 3 Department of Precision Medicine, Kyung Hee University College of Medicine, Seoul, South Korea; 4 Department of Applied Information Engineering, Yonsei University, Seoul, South Korea; 5 Department of Epidemiology, Harvard T.H. Chan School of Public Health, Boston, MA USA; 6 Department of Electronics and Information Convergence Engineering, Kyung Hee University, Yongin, South Korea; 7 Department of Neurology, Kyung Hee University Medical Center, Kyung Hee University College of Medicine, Seoul, South Korea; 8 Department of Health Economics and Health Services Research, University Medical Center Hamburg-Eppendorf, Hamburg Center for Health Economics, Hamburg, Germany; 9 Department of Pediatrics, Kyung Hee University Medical Center, Kyung Hee University College of Medicine, 23 Kyungheedae-Ro, Dongdaemun-Gu, Seoul, 02447 South Korea; 10 Centre for Health, Performance and Wellbeing, Anglia Ruskin University, Cambridge, CB1 1PT UK; 11 Department of Public Health, Faculty of Medicine, Biruni University, Istanbul, Turkey

**Keywords:** Adolescent, Epidemiology, Suicidal ideation, Trend, WHO, Diseases, Health care, Medical research, Risk factors

## Abstract

Although suicidal ideation is a significant issue among adolescents, previous studies have largely relied on cross-sectional data collected at a single time point or have been limited to individual countries, providing limited insight into temporal trends across diverse populations. Therefore, we aimed to examine temporal trends in suicidal ideation among adolescents across 23 countries. We analyzed data from the Global School-Based Student Health Survey (2003–2021), which included adolescents aged 13–15 years in 23 countries. Each participant participated in multiple surveys, and survey years varied by country. Prior to trend estimation, we compared linear and quadratic fits where more than three surveys were available to identify near-linear patterns. Temporal trend was quantified as the average annual percentage change (AAPC), which was calculated by weighted log-linear regression on the log prevalence rates of the survey years, applied separately to boys and girls. The study analyzed 185,941 school-attending adolescents (46.45% male) across 23 countries. The prevalence of suicidal ideation showed significant upward trends in six countries: Myanmar (AAPC, 32.04%/year; 2007–2016), Guyana (AAPC, 8.88%/year; 2010–2014), Saint Vincent and the Grenadines (AAPC, 3.87%/year; 2007–2018), Mongolia (AAPC, 3.67%/year; 2010–2013), Bolivia (AAPC, 3.02%/year; 2012–2018), and Seychelles (AAPC, 2.54%/year; 2007–2015). Conversely, five countries exhibited significant declines, including Benin (AAPC, -8.60%/year; 2009–2016), Kuwait (AAPC, -6.40%/year; 2011–2015), and the Maldives (AAPC, -4.33%/year; 2009–2014). Sex-specific differences in trends were nominally significant (p < 0.05) in six countries—Benin, Kuwait, Argentina, Saint Vincent and the Grenadines, Thailand, and Guyana—but only two (Saint Vincent and the Grenadines and Thailand) remained statistically significant after Bonferroni correction. In five of these countries (excluding Guyana), girls exhibited more unfavorable patterns, showing either a greater increase or a smaller decrease in suicidal ideation compared to boys. This study highlights divergent trends in adolescent suicidal ideation across 23 countries, with rising prevalence in some regions and notable sex differences. The findings underscore the need for continued surveillance and context-specific mental health interventions.

**Supplementary Information:**

The online version contains supplementary material available at 10.1038/s41598-025-19158-5.

## Introduction

Adolescence is a critical period characterized by rapid physiological, psychological, and social development, during which many psychological problems first emerge^1^. Mental disorders during this stage can lead to numerous adverse outcomes, with suicidal behavior being among the most fatal^1^. According to the World Health Organization (WHO), suicide ranks as the second leading global cause of death for young people aged 10–24 years, underscoring the severity of this public health issue^2^. Suicidal ideation, which refers to severe thoughts about taking one’s own life, is particularly concerning in adolescents^3^. Research estimates that approximately one-third of adolescents who experience suicidal thoughts eventually attempt suicide, highlighting the need for early detection and timely public health intervention^4–6^..

Several previous studies have examined the cross-sectional prevalence of suicidal ideation across multiple countries^7^, as well as within individual nations^8,9^. A systematic review conducted a meta-analysis on younger children aged 6–12 years, reporting a global prevalence of 7.5% (95% confidence interval [CI], 5.9–9.6) for suicidal ideation^10^. However, limited evidence exists on how suicidal ideation among adolescents has changed over time across multiple countries. Most available studies provide only single-timepoint estimates, making it difficult to assess whether rates are increasing or decreasing and to what extent these trends differ by region or sex^11^. This gap in the literature restricts our ability to design data-driven, regionally tailored suicide prevention policies.

To address this gap, we used data from the WHO’s Global School-based Student Health Survey (GSHS) to examine temporal trends in suicidal ideation among adolescents aged 13–15 years across 23 countries^12^. By identifying patterns over time and exploring sex-based differences, this study aims to support evidence-based efforts to reduce adolescent suicidal ideation at both national and global levels.

## Methods

### Survey and participants

The study aimed to raise awareness of adolescents’suicidal ideation, identify trends in suicidal ideation among 185,941 adolescents in 23 countries around the world, and report on measures that can be implemented at the national level to prevent adolescents’suicidal ideation^13^. We analyzed data from 23 countries (African region [AFR]; Benin and Seychelles; Region of the Americas [AMR]; Argentina, Bolivia, Guatemala, Guyana, Jamaica, Saint Vincent and the Grenadines, Suriname, and Trinidad and Tobago; Eastern Mediterranean region [EMR]; Jordan, Kuwait, Lebanon, Morocco, and United Arab Emirates; South-East Asian region [SEAR]: Maldives, Myanmar, Sri Lanka, and Thailand; Western pacific region [WPR]: Mongolia, Philippines, Samoa, and Vanuatu) available to the public from the GSHS^13^. All GSHS surveys were approved, in each country, by both an institutional review board or ethics committee and a national government administration. Informed consent was obtained as appropriate from the students, parents, and/or school officials. Ethical considerations were upheld, adhering to the Declaration of Helsinki.

The GSHS was executed by WHO alongside the United States Centers for Disease Control and Prevention and various United Nations allies. This survey aimed to gather information on the health behaviors and protective measures among adolescents across the globe, featuring ten principal questionnaire modules, including one focused on suicidal ideation^14,15^. The survey was adapted into local languages, and there may be some differences in the wording and structure of the questionnaire across countries and years; however, to minimize heterogeneity across instruments, we only used the Standard Variable Names defined in the GSHS survey^16^.

The method of selecting participants involved a uniform two-step scientific sampling methodology. Initially, schools were chosen based on a probability proportional to size approach. Subsequently, classrooms with students aged 13–15 years were randomly selected within these schools. However, all students in these classrooms were invited to partake, not just those within the specific age bracket. Consequently, initial data collection included ages beyond the targeted 13–15 range, but these individuals were not considered in the final analysis. Authorization for conducting the GSHS was secured from the national government administrations and ethics committees or institutional review boards in each country^16^. To maintain the anonymity and voluntary nature of the study, specific protocols were put in place. Furthermore, the study utilized statistical adjustments to address potential biases due to non-response and differential selection probabilities^17^.

To identify temporal trends, we included all GSHS countries whose nationally representative datasets contained the standard suicidal-ideation item and had been surveyed on at least two years between 2003 and 2021^18^. This study included an analysis of 23 countries in total. Table 1provides comprehensive details about the characteristics of each country in the year of the survey. This information encompasses the sample size, response rate, the proportion of male participants, and the income level of the country. All students were given an information sheet about the GSHS and a consent form to secure written permission from their parents or guardians before the start of the study^18^.

**Table 1:** Characteristics of surveys on suicidal ideation extracted from the WHO GSHS database (2003–2021).

Country	Survey year	Sample size, n	Response rate, %*	Boys, %	Country income level**
**African Region (AFR)**					
Benin	2009	1095	99.91	63.35	LIC
	2016	686	99.71	45.91	LIC
Seychelles	2007	928	97.52	46.08	UMIC
	2015	1518	95.32	45.54	HIC
**Region of the Americas (AMR)**					
Argentina	2007	1512	99.01	47.23	UMIC
	2012	20,890	97.40	47.03	UMIC
	2018	36,332	97.49	48.18	UMIC
Bolivia	2012	2678	98.47	49.94	LMIC
	2018	3851	93.20	50.85	LMIC
Guatemala	2009	4283	98.58	45.05	LMIC
	2015	3444	95.64	48.12	LMIC
Guyana	2010	1953	97.75	44.63	LMIC
	2014	1052	97.81	39.65	LMIC
Jamaica	2010	1192	94.63	48.40	UMIC
	2017	1054	95.92	45.20	UMIC
Saint Vincent and the Grenadines	2007	1029	95.34	45.97	UMIC
	2018	1024	98.14	45.67	UMIC
Suriname	2009	956	99.27	47.21	UMIC
	2016	1322	97.66	45.47	UMIC
Trinidad and Tobago	2011	1836	96.73	54.45	HIC
	2017	2153	97.21	47.68	HIC
**Eastern Mediterranean Region (EMR)**					
Jordan	2004	1844	95.28	44.56	LMIC
	2007	1631	93.99	54.86	LMIC
Kuwait	2011	2254	99.25	49.13	HIC
	2015	1990	91.81	45.21	HIC
Lebanon	2005	3754	98.75	46.26	UMIC
	2011	1714	98.31	46.35	UMIC
	2017	2751	97.42	40.49	UMIC
Morocco	2006	1866	97.91	48.49	LMIC
	2010	1997	98.30	51.50	LMIC
	2016	3262	94.51	51.05	LMIC
United Arab Emirates	2005	10,669	97.36	48.59	HIC
	2010	2179	97.84	39.49	HIC
	2016	3190	56.93	48.13	HIC
**South-East Asian Region (SEAR)**					
Maldives	2009	1971	94.11	43.61	LMIC
	2014	1765	94.22	40.59	UMIC
Myanmar	2007	1983	99.95	48.54	LIC
	2016	1957	99.59	46.84	LMIC
Sri Lanka	2008	2260	97.57	44.72	LMIC
	2016	2196	98.82	43.55	LMIC
Thailand	2008	2,223	98.47	49.57	LMIC
	2015	3492	95.13	45.79	UMIC
	2021	3621	97.40	44.97	UMIC
**Western Pacific Region (WPR)**					
Mongolia	2010	3129	98.85	44.00	LMIC
	2013	3113	98.88	48.28	LMIC
Philippines	2003	4160	97.14	39.57	LMIC
	2007	3433	99.07	40.08	LMIC
	2011	3640	98.63	41.14	LMIC
	2015	5635	97.66	43.63	LMIC
	2019	6469	96.78	44.63	LMIC
Samoa	2011	2116	80.77	38.33	LMIC
	2017	960	94.79	32.20	LMIC
Vanuatu	2011	704	98.15	40.81	LMIC
	2016	1225	97.14	40.50	LMIC

Abbreviations: CI, confidence interval; GSHS, Global School-based Student Health Survey; HIC, high-income countries; LIC, low-income countries; LMIC, lower-middle income countries; UMIC, upper-middle income countries; WHO, World Health Organization; WHO FCTC, World Health Organization Framework Convention on Tobacco Control.

* Response rate is for the suicidal ideation questions. Data are for participants aged 13–15 years.

**Country income level was categorized by World bank income category.

### Suicidal ideation

Our study focused on serious suicidal ideation rather than actual suicide attempts due to the limited number of countries in the GSHS dataset that included information on actual suicide attempts^19^. This decision was based on the premise that suicidal ideations are considered a significant precursor to potential suicide attempts, offering essential insights into an individual’s mental state before actual suicidal actions are taken^20,21^. Furthermore, suicidal ideation acts as a crucial indicator of mental health crises, providing a wider basis for understanding and intervening in the early stages of such crises, and is recognized as a more common phenomenon than actual suicide attempts, thus serving as an important indicator of mental health^20^. Suicidal ideation was assessed through the question, “During the past 12 months, did you ever seriously consider attempting suicide?”. To improve the accuracy of our analysis, we concentrated on participants aged 13–15 years. This focus was due to the majority of students surveyed falling within this age group and the lack of detailed age information for individuals outside of this range. Responses to this item were treated with a complete-case approach: records with a missing answer were excluded from the analysis, while the original GSHS sampling weights—which already incorporate school- and student-level non-response adjustments—were retained without further modification^12^.

### Statistical analysis

We calculated the weighted prevalence of serious suicidal ideation in the preceding 12 months and its 95% CI for the total sample and separately for boys and girls. Temporal change in each country was expressed as the average annual percent change (AAPC)^22^. When three or more survey waves were available, the AAPC and its confidence interval were derived from a log-linear regression of weighted prevalence on calendar year^22^. When only two waves were available, the AAPC and its variance were calculated directly from the log-difference between the first and final surveys. For each estimate, a two-sided P-value was obtained, and all P-values were adjusted for multiple testing with the Bonferroni procedure; adjusted values below 0.05 were deemed statistically significant.

To identify that a straight-line specification was appropriate, a linear model was formally compared with a quadratic alternative in every country that provided at least three survey waves. Model fit was evaluated with the Akaike and Bayesian information criteria, and the curvature term was tested for significance. A trend was classified as non-linear when the quadratic term reached a P-value < 0.05 or when the quadratic model provided a lower information-criterion value^23^. The full diagnostic results are reported in Table S1, and the corresponding residual plots are presented in Figure S1. All statistical inferences were defined as significant at a two-sided P-value less than 0.05. Statistical analyses were performed using R (version 4.3.2; R Foundation, Vienna, Austria) and Python (version 3.11.4; Python Software Foundation).

## Results

Based on data from the GSHS conducted between 2003 and 2021, 23 countries across five WHO regions, each with data from two or more survey years, were included in the assessment of trends in suicidal ideation. Table 1 provides a detailed overview of the surveys, including information such as survey year, sample sizes, response rates, the percentage of boys participating, and the income levels of the countries categorized by the World Bank income classification. Except for the United Arab Emirates in 2016 (56.93%), all surveys recorded high response rates of over 90%, regardless of the survey year.

**Figure 1:**
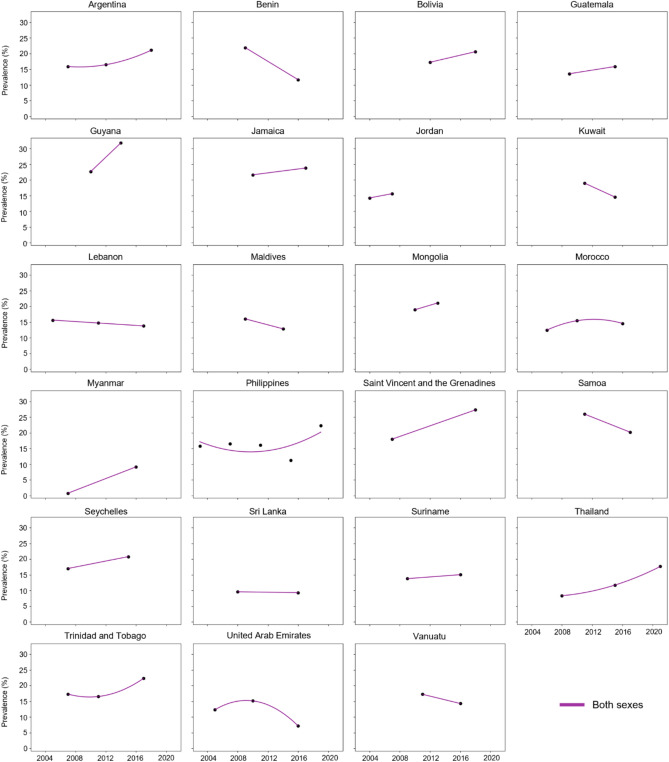
Temporal change of suicidal ideation among adolescents aged 13–15 years across 23 countries from 2003 to 2021.

Figure 1 visualizes the trend in the prevalence of suicidal ideation over time. Table 2 shows suicidal ideation prevalence by country, year, and sex, along with the AAPC and p-values. In the AFR, Benin showed a significant decrease in the overall prevalence of suicidal ideation from 21.91% (95% CI, 19.37 to 24.44) in 2009 to 11.67% (95% CI, 9.01 to 14.33) in 2016 (AAPC, −8.60%/year [95% CI, −11.88 to −5.20]). Conversely, Seychelles experienced an increase in prevalence from 17.03% (95% CI, 14.57 to 19.49) in 2007 to 20.82% (95% CI, 18.74 to 22.91) in 2015 (AAPC, 2.54%/year [95% CI, 0.32 to 4.82]). The AMR generally presented a modest increase in prevalence. However, significant increases were observed in Bolivia (17.24% [95% CI, 15.68 to 18.80] in 2012; 20.61% [95% CI, 19.28 to 21.94] in 2018; AAPC, 3.02%/year [95% CI, 1.13 to 4.95]), Guyana (22.66% [95% CI, 20.70 to 24.61] in 2010; 31.84% [95% CI, 28.92 to 34.76] in 2014; AAPC, 8.88%/year [95% CI, 5.51 to 12.36]) and Saint Vincent and the Grenadines (17.98% [95% CI, 15.52 to 20.45] in 2007; 27.30% [95% CI, 24.52 to 30.07] in 2018; AAPC, 3.87%/year [95% CI, 2.27 to 5.49]). Meanwhile, in the EMR, Kuwait exhibited a steep decline in suicidal ideation prevalence, dropping from 18.98% (95% CI, 17.32 to 20.64) in 2011 to 14.57% (95% CI, 12.96 to 16.18) in 2015 (AAPC, −6.40%/year [95% CI, −9.65 to −3.04]). Similarly, Lebanon showed a moderate but significant decrease from 15.61% (95% CI, 14.44 to 16.79) in 2005 to 13.76% (95% CI, 12.26 to 15.26) in 2017, corresponding to an AAPC of −1.05%/year (95% CI, −1.80 to −0.29). In the SEAR, a dramatic increase in prevalence was observed in Myanmar (0.75% [95% CI, 0.35 to 1.15] in 2007; 9.14% [95% CI, 7.80 to 10.47] in 2016; AAPC, 32.04/year [95% CI, 24.21 to 40.37]), while Maldives (16.03% [95% CI, 14.24 to 17.81] in 2009; 12.85% [95% CI, 11.04 to 14.65] in 2014; AAPC, −4.33%/year [95% CI, −7.70 to 0.83]) showed decreasing trends. The WPR displayed heterogeneous trends. The Philippines experienced a decrease in prevalence from 2003 (15.76% [95% CI, 14.29 to 17.23]) to 2015 (11.18% [95% CI, 10.23 to 12.13]) followed by a reversal from 2015 to 2019 (22.29% [95% CI, 21.14 to 23.44]; AAPC, 0.76%/year [95% CI, −6.03 to 8.03]). Samoa experienced a significant decrease of prevalence from 2011 (25.95% [95% CI, 24.02 to 27.87]) to 2017 (20.21% [95% CI, 17.38 to 23.03]; AAPC, −4.08%/year [95% CI, −6.58 to −1.52]), while increase was observed in Mongolia (AAPC, 3.67%/year [95% CI, 0.22 to 7.23]).

**Table 2:** The trend of suicidal ideation in young adolescents aged 13–15 years by WHO region and sex.

Country	Survey year	Total			Boys			Girls		
		Prevalence (95% CI)	AAPC (95% CI)	P-value	Prevalence (95% CI)	AAPC (95% CI)	P-value	Prevalence (95% CI)	AAPC (95% CI)	P-value
**African Region (AFR)**										
Benin^a^	2009	21.91 (19.37 to 24.44)	**−8.60 (−11.88 to −5.20)**	** < 0.001**^c^	23.22 (19.96 to 26.48)	**−12.08 (−16.74 to −7.17)**	** < 0.001**^c^	19.40 (15.44 to 23.36)	−2.83 (−7.12 to 1.67)	0.214
	2016	11.67 (9.01 to 14.33)			9.43 (6.09 to 12.76)			15.87 (12.03 to 19.71)		
Seychelles	2007	17.03 (14.57 to 19.49)	**2.54 (0.32 to 4.82)**	**0.025**	14.85 (11.45 to 18.25)	0.02 (−3.56 to 3.73)	0.993	19.19 (15.68 to 22.70)	**4.03 (1.25 to 6.90)**	**0.004**
	2015	20.82 (18.74 to 22.91)			14.87 (12.19 to 17.55)			26.33 (23.24 to 29.42)		
**Region of the Americas (AMR)**										
Argentina^a^	2007	15.88 (13.91 to 17.86)	2.66 (−9.31 to 16.21)	0.227	12.50 (9.90 to 15.10)	0.29 (−19.35 to 24.71)	0.893	18.68 (15.79 to 21.57)	3.97 (−2.75 to 11.15)	0.085
	2012	16.45 (15.53 to 17.38)			10.72 (9.54 to 11.89)			21.55 (20.19 to 22.91)		
	2018	21.07 (20.25 to 21.90)			12.78 (11.78 to 13.78)			28.58 (27.33 to 29.83)		
Bolivia	2012	17.24 (15.68 to 18.80)	**3.02 (1.13 to 4.95)**	**0.002**	11.77 (9.84 to 13.70)	**4.00 (0.65 to 7.47)**	**0.019**	22.45 (20.06 to 24.83)	**2.50 (0.27 to 4.79)**	**0.028**
	2018	20.61 (19.28 to 21.94)			14.90 (13.27 to 16.53)			26.04 (23.97 to 28.10)		
Guatemala	2009	13.58 (12.33 to 14.84)	2.63 (−0.41 to 5.77)	0.091	9.63 (8.05 to 11.21)	2.38 (−2.96 to 8.02)	0.389	17.79 (15.89 to 19.68)	2.59 (−0.95 to 6.26)	0.153
	2015	15.87 (13.41 to 18.34)			11.09 (8.02 to 14.16)			20.74 (16.97 to 24.52)		
Guyana^a^	2010	22.66 (20.70 to 24.61)	**8.88 (5.51 to 12.36)**	** < 0.001**^c^	16.31 (13.75 to 18.86)	**14.43 (8.22 to 20.99)**	** < 0.001**^c^	28.57 (25.72 to 31.42)	**5.36 (1.56 to 9.30)**	**0.005**
	2014	31.84 (28.92 to 34.76)			27.96 (23.52 to 32.40)			35.20 (31.40 to 39.00)		
Jamaica	2010	21.66 (18.45 to 24.87)	1.36 (−1.30 to 4.10)	0.320	18.46 (14.03 to 22.89)	−1.52 (−5.89 to 3.06)	0.509	24.75 (20.15 to 29.35)	3.02 (−0.27 to 6.41)	0.072
	2017	23.81 (21.12 to 26.50)			16.59 (13.13 to 20.04)			30.47 (26.51 to 34.44)		
Saint Vincent and the Grenadines^b^	2007	17.98 (15.52 to 20.45)	**3.87 (2.27 to 5.49)**	** < 0.001**^c^	15.54 (12.18 to 18.90)	−0.61 (−3.37 to 2.24)	0.673	20.19 (16.61 to 23.76)	**5.98 (4.00 to 7.99)**	** < 0.001**^c^
	2018	27.30 (24.52 to 30.07)			14.53 (11.30 to 17.77)			38.23 (34.12 to 42.34)		
Suriname	2009	13.80 (11.60 to 16.00)	1.26 (−1.69 to 4.31)	0.406	11.23 (8.30 to 14.15)	−1.42 (−6.38 to 3.82)	0.589	15.95 (12.74 to 19.16)	2.73 (−0.91 to 6.51)	0.144
	2016	15.07 (13.06 to 17.07)			10.16 (7.61 to 12.71)			19.26 (16.31 to 22.21)		
Trinidad and Tobago	2007	17.29 (15.07 to 19.51)	2.78 (−17.05 to 27.35)	0.351	13.40 (10.62 to 16.19)	1.50 (−4.48 to 7.85)	0.198	21.17 (17.73 to 24.61)	3.39 (−24.26 to 41.14)	0.403
	2011	16.51 (14.45 to 18.58)			13.61 (11.03 to 16.19)			19.30 (16.08 to 22.52)		
	2017	22.31 (20.28 to 24.34)			15.47 (12.88 to 18.06)			28.74 (25.73 to 31.75)		
**Eastern Mediterranean Region (EMR)**										
Jordan	2004	14.29 (12.65 to 15.94)	3.05 (−2.41 to 8.82)	0.279	12.31 (10.02 to 14.59)	4.79 (−3.45 to 13.74)	0.263	16.11 (13.77 to 18.45)	1.74 (−5.43 to 9.46)	0.643
	2007	15.64 (13.83 to 17.45)			14.16 (11.88 to 16.45)			16.97 (14.17 to 19.76)		
Kuwait^a^	2011	18.98 (17.32 to 20.64)	**−6.40 (−9.65 to −3.04)**	** < 0.001**^c^	18.39 (16.04 to 20.74)	**−10.74 (−15.62 to −5.58)**	** < 0.001**^c^	19.62 (17.27 to 21.96)	−3.01 (−7.30 to 1.47)	0.184
	2015	14.57 (12.96 to 16.18)			11.67 (9.51 to 13.83)			17.36 (15.01 to 19.71)		
Lebanon	2005	15.61 (14.44 to 16.79)	**−1.05 (−1.80 to −0.29)**	**0.036**	13.73 (12.09 to 15.38)	0.14 (−14.59 to 17.42)	0.928	17.37 (15.70 to 19.03)	−2.04 (−13.70 to 11.20)	0.287
	2011	14.75 (12.78 to 16.72)			12.16 (9.36 to 14.96)			17.02 (14.30 to 19.74)		
	2017	13.76 (12.26 to 15.26)			13.97 (11.73 to 16.21)			13.56 (11.55 to 15.57)		
Morocco	2006	12.43 (10.89 to 13.97)	1.40 (−18.72 to 26.51)	0.570	10.90 (8.81 to 12.99)	1.39 (−4.89 to 8.09)	0.222	14.13 (11.89 to 16.37)	1.35 (−29.48 to 45.66)	0.720
	2010	15.44 (13.83 to 17.04)			12.07 (10.05 to 14.09)			19.40 (16.90 to 21.91)		
	2016	14.58 (13.14 to 16.02)			12.59 (10.67 to 14.52)			16.70 (14.55 to 18.85)		
United Arab Emirates	2005	12.30 (11.59 to 13.02)	−5.07 (−48.11 to 73.65)	0.471	12.13 (11.14 to 13.12)	−5.64 (−44.66 to 60.89)	0.399	12.47 (11.44 to 13.51)	−4.57 (−49.21 to 79.29)	0.519
	2010	15.12 (13.53 to 16.72)			13.70 (11.36 to 16.05)			16.07 (13.92 to 18.21)		
	2016	7.14 (6.16 to 8.11)			6.56 (5.20 to 7.92)			7.67 (6.29 to 9.06)		
**South-East Asian Region (SEAR)**										
Maldives	2009	16.03 (14.24 to 17.81)	**−4.33 (−7.70 to −0.83)**	**0.016**	14.98 (12.34 to 17.61)	−5.03 (−10.43 to 0.69)	0.084	16.98 (14.58 to 19.38)	−3.73 (−7.88 to 0.61)	0.091
	2014	12.85 (11.04 to 14.65)			11.57 (8.86 to 14.28)			14.04 (11.67 to 16.42)		
Myanmar	2007	0.75 (0.35 to 1.15)	**32.04 (24.21 to 40.37)**	** < 0.001**^c^	0.83 (0.23 to 1.43)	**27.93 (17.51 to 39.28)**	** < 0.001**^c^	0.67 (0.15 to 1.19)	**35.76 (24.22 to 48.38)**	** < 0.001**^c^
	2016	9.14 (7.80 to 10.47)			7.59 (5.79 to 9.39)			10.51 (8.57 to 12.45)		
Sri Lanka	2008	9.60 (8.35 to 10.84)	−0.32 (−2.61 to 2.02)	0.786	10.29 (8.38 to 12.21)	−0.32 (−3.60 to 3.08)	0.853	8.90 (7.30 to 10.49)	−0.29 (−3.44 to 2.96)	0.859
	2016	9.35 (8.10 to 10.60)			10.04 (8.10 to 11.97)			8.69 (7.10 to 10.29)		
Thailand^b^	2008	8.37 (7.17 to 9.57)	5.89 (−1.41 to 13.74)	0.062	9.24 (7.52 to 10.97)	1.48 (−9.21 to 13.43)	0.342	7.52 (5.86 to 9.18)	9.37 (−7.10 to 28.77)	0.091
	2015	11.75 (10.32 to 13.17)			11.28 (9.28 to 13.29)			12.23 (10.21 to 14.25)		
	2021	17.67 (16.05 to 19.29)			11.14 (9.15 to 13.13)			24.28 (21.86 to 26.71)		
**Western Pacific Region (WPR)**										
Mongolia	2010	18.93 (17.53 to 20.34)	**3.67 (0.22 to 7.23)**	**0.037**	15.12 (13.18 to 17.05)	2.61 (−3.15 to 8.71)	0.383	22.41 (20.43 to 24.39)	**4.86 (0.68 to 9.23)**	**0.022**
	2013	21.09 (19.63 to 22.56)			16.33 (14.43 to 18.24)			25.84 (23.66 to 28.02)		
Philippines	2003	15.76 (14.29 to 17.23)	0.76 (−6.03 to 8.03)	0.753	16.97 (14.59 to 19.35)	−0.65 (−8.36 to 7.71)	0.815	14.93 (13.06 to 16.80)	1.92 (−5.23 to 9.61)	0.466
	2007	16.53 (15.09 to 17.98)			12.52 (10.49 to 14.55)			19.74 (17.74 to 21.74)		
	2011	16.06 (14.60 to 17.52)			11.34 (9.35 to 13.33)			20.45 (18.43 to 22.46)		
	2015	11.18 (10.23 to 12.13)			9.01 (7.74 to 10.28)			13.21 (11.81 to 14.60)		
	2019	22.29 (21.14 to 23.44)			17.57 (16.01 to 19.12)			26.72 (25.06 to 28.38)		
Samoa	2011	25.95 (24.02 to 27.87)	**−4.08 (−6.58 to −1.52)**	**0.002**	27.98 (24.88 to 31.07)	**−5.55 (−9.62 to −1.30)**	**0.011**	24.19 (21.78 to 26.60)	−2.72 (−5.74 to 0.39)	0.086
	2017	20.21 (17.38 to 23.03)			19.86 (15.09 to 24.62)			20.50 (17.20 to 23.79)		
Vanuatu	2011	17.22 (14.09 to 20.36)	−3.67 (−8.09 to 0.96)	0.118	17.03 (12.11 to 21.95)	0.73 (−6.14 to 8.11)	0.839	17.39 (13.40 to 21.38)	**−8.35 (−13.97 to −2.36)**	**0.007**
	2016	14.28 (12.16 to 16.40)			17.66 (14.06 to 21.27)			11.24 (8.79 to 13.70)		

Abbreviations: AAPC, average annual percent change; CI, confidence interval; WHO, World Health Organization.

The numbers in bold indicate significant differences (P-value < 0.05).

^a^ indicates a statistically significant difference in AAPC between boys and girls.

^b^ indicates a statistically significant difference in AAPC between boys and girls even after Bonferroni correction.

^c^ indicates a statistically significant in AAPC even after Bonferroni correction.

**Figure2:**
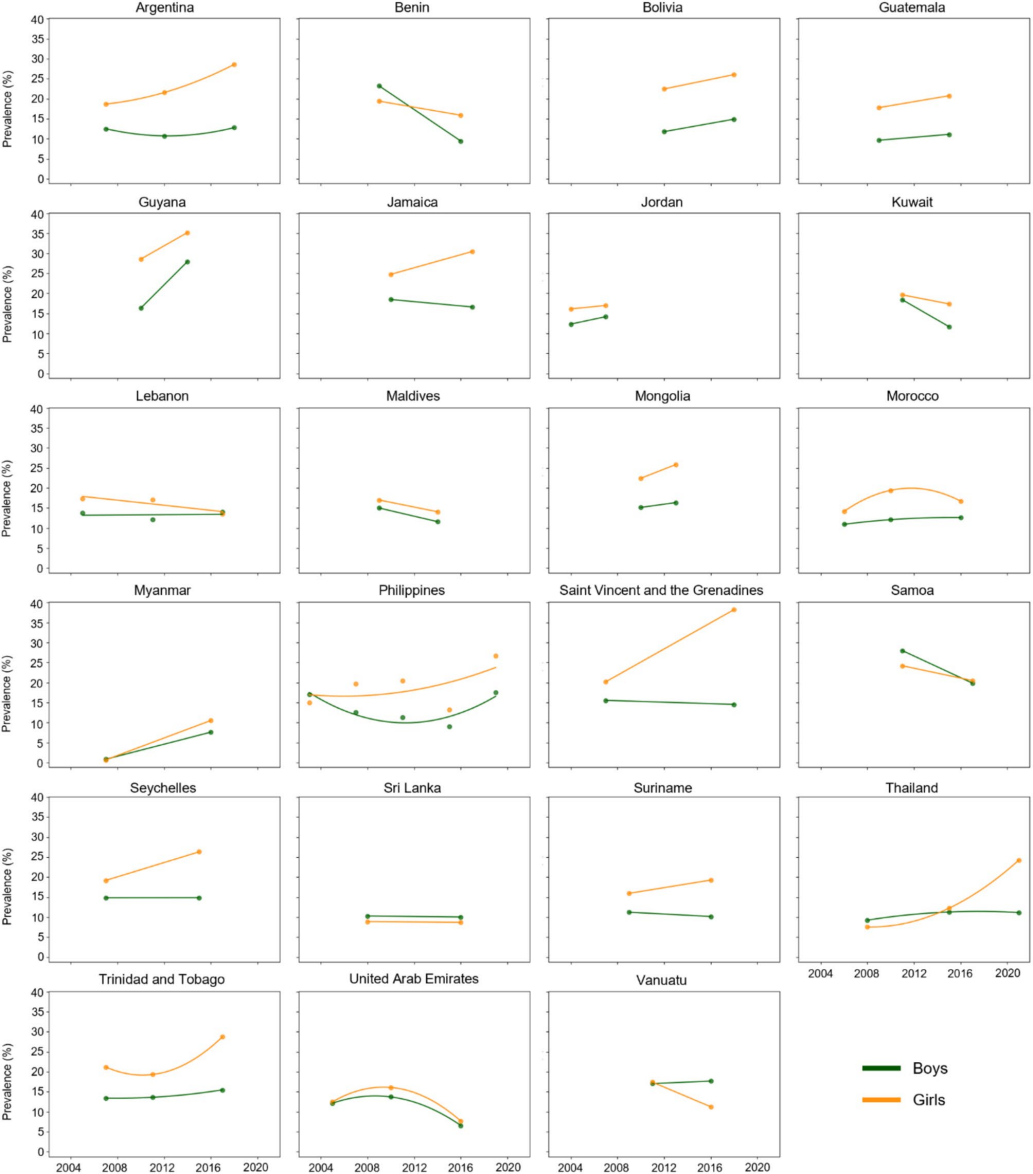
Temporal change of suicidal ideation among boys and girls aged 13–15 years across 23 countries from 2003 to 2021.

Figure 2 presents the trends in suicidal ideation prevalence stratified by sex. Among the 23 countries analyzed, six countries exhibited statistically significant sex differences in AAPC values at a nominal significance level (p < 0.05): Benin (AFR); Argentina, Guyana, Saint Vincent and the Grenadines (AMR); Kuwait (EMR); and Thailand (SEAR). However, only two of these countries, Saint Vincent and the Grenadines and Thailand, retained statistical significance after Bonferroni correction. Specifically, in Benin (AAPC, boys: −12.08%/year [95% CI, −16.74 to −7.17]; girls: −2.83%/year [95% CI, −7.12 to 1.67]) and Kuwait (AAPC, boys: −10.74%/year [95% CI, −15.62 to −5.58]; girls: −3.01%/year [95% CI, −7.30 to 1.47]), boys showed notably larger declines in suicidal ideation prevalence compared to girls. Conversely, in Argentina (AAPC, boys: 0.29%/year [95% CI, −19.35 to 24.71]; girls: 3.97%/year [95% CI, −2.75 to 11.15]), Saint Vincent and the Grenadines (AAPC, boys: −0.61%/year [95% CI, −3.37 to 2.24]; girls: 5.98%/year [95% CI, 4.00 to 7.99]), and Thailand (AAPC, boys: 1.48%/year [95% CI, −9.21 to 13.43]; girls: 9.37%/year [95% CI, −7.10 to 28.77]), girls exhibited a more pronounced increase in prevalence than boys. In contrast, in Guyana (AAPC, boys: 14.43%/year [95% CI, 8.22 to 20.99]; girls: 5.36%/year [95% CI, 1.56 to 9.30]), boys experienced a greater increase in prevalence compared to girls.

**Fig. 3:**
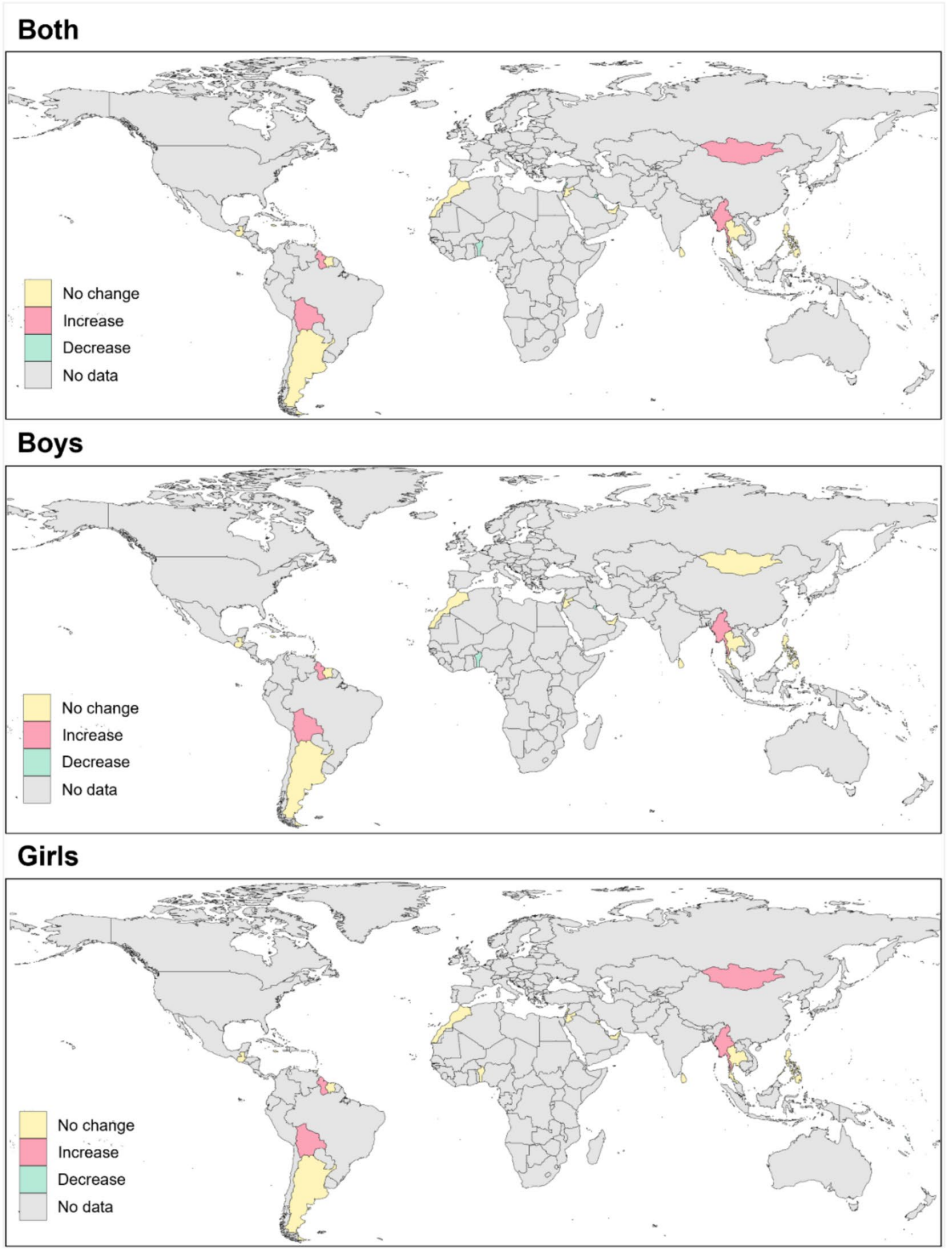
Color-coded world map displaying global trends (increase, decrease, and no change) in suicidal ideation among young adolescents aged 13–15 years in 23 countries from 2003 to 2021. The maps were created using R (version 4.3.2; R Foundation for Statistical Computing, Vienna, Austria; https://www.r-project.org/).

Figure 3 presents a color-coded map illustrating sex-specific trends in the prevalence of adolescent suicidal ideation, based on the direction and statistical significance of the AAPC. Distinct regional patterns emerged: Latin American and Caribbean countries, particularly Guyana and Saint Vincent and the Grenadines, exhibited significant increases, while Middle Eastern countries such as Kuwait and Lebanon showed notable decreases. When disaggregated by sex, statistically significant increases were observed among boys in three countries and decreases in another three. Among girls, six countries showed significant increases, whereas only one country exhibited a decrease.

## Discussion

### Key finding

This study presents a global assessment of temporal trends in the prevalence of suicidal ideation among adolescents, addressing a critical gap in existing literature. An increasing trend was identified in six countries, with the most substantial rise observed in Myanmar, followed by Guyana, Saint Vincent and the Grenadines, Mongolia, Bolivia, and Seychelles. Conversely, a declining trend was noted in five countries: Benin, Kuwait, the Maldives, Samoa, and Lebanon. Sex-specific patterns were detected in six countries based on nominal statistical significance (p < 0.05), with differences remaining significant after Bonferroni correction in two of them. In particular, Argentina, Saint Vincent and the Grenadines, and Thailand exhibited a more pronounced increase in suicidal ideation among girls, while Benin and Kuwait showed a greater decrease among boys. The rising trends in several countries and the presence of sex-based disparities highlight a pressing need for targeted mental health interventions. Future public health strategies may benefit from examining and adopting successful approaches implemented in countries where suicidal ideation has decreased over time.

### Interpretation of findings

Previous studies report considerable variability in the prevalence of suicidal ideation across different income levels, countries, and sexes^24^, and suicidal ideation is most common among adolescents^24,25^. The near-equal number of countries exhibiting increases (six countries) and decreases (five countries) in suicidal ideation suggests heterogeneity in temporal patterns, highlighting the need to consider country-specific factors when developing mental health strategies.

While the precise causes of the increasing trends are challenging to pinpoint because of the lack of existing literature, they may be associated with several factors such as social structures, social values, economic turmoil, or environmental conditions (such as ongoing conflicts or social unrest)^24^. For example, in Myanmar, the breakdown of the ceasefire in Kachin State in 2011 led to renewed conflict and severe mental health issues among the affected population^26^. In addition, in 2012 and 2017, mass violence compelled hundreds of thousands of ethnic minorities (Rohingya people) to flee to Bangladesh, facing harsh refugee conditions^27^. These factors May help contextualize the presence of mental health issues among adolescents in conflict zones, which could relate to a 12-fold increase in suicidal ideation in Myanmar^26,27^. Similarly, in Guyana, the increasing rates of suicidal ideation among adolescents May be influenced by broader sociocultural factors. Between 2010 and 2014, the country experienced a series of destabilizing events, including severe nationwide flooding in 2013 that displaced Many residents and the prorogation of Parliament in 2014,which raised public concerns about democratic backsliding^28,29^. Although direct causal associations between these events and mental health outcomes cannot be identified, they occurred during a period of rising social and economic instability, which may help contextualize the upward trend. In addition, existing literature has identified widespread interpersonal violence and persistent ethnic and class-based tensions in Guyana as potential risk factors for poor mental health outcomes^30,31^. Additionally, Thailand was the only country in our study where the survey period extended into the COVID-19 pandemic, and the prevalence of suicidal ideation among adolescents in Thailand increased by more than 6% points between 2015 and 2021. This trend may reflect the mental health impact of the pandemic, which was characterized by widespread fear, uncertainty, and social disruption^32^.

Conversely, several countries exhibited a notable decline in suicidal ideation. While direct evidence explaining the causal mechanisms behind these declines remains scarce, prior research suggests that improved mental health policies, the stabilization of previously unstable sociopolitical environments, and reduced stigma toward individuals with mental health conditions may have contributed to the observed trends^33,34^. For example, in Lebanon, the government launched the National Mental Health Program (NMHP) in 2014, initiating structured public mental health reform with support from WHO and the United Nations International Children’s Emergency Fund. The NMHP introduced the Mental Health and Substance Use Strategy 2015–2020, aiming to expand mental health services, reduce stigma, and promote cross-sectoral collaboration across health, education, and social protection systems^35^. Importantly, these reforms occurred during a period of gradual recovery following decades of civil war, political assassinations, and regional instability. While Lebanon continued to face challenges, including the large influx of Syrian refugees from 2011 onwards, the relative post-conflict stabilization of Lebanese society may have created a more conducive environment for implementing public mental health initiatives^35^. This broader social and institutional stability likely played a supportive role in reducing mental health burdens and suicidal ideation, particularly among adolescents.

Temporal trends in sex differences in suicidal ideation were observed in six countries, with five countries showing an adverse trend among girls compared to boys. This finding is more surprising than other studies that report a higher prevalence of suicidal ideation among girls (15.1% in boys and 18.5% in girls)^10,36^. This could imply that the issue is likely to become more severe among girls in the future. The reasons remain elusive and could be attributed to various socio-cultural characteristics or biopsychosocial factors. Compared to boys, girls are more likely to exhibit mental health issues such as depression and stress-related disorders^37^. Furthermore, girls in low- and middle-income countries (LMICs) are more likely to experience sex inequity and various forms of maltreatment, particularly domestic violence and childhood sexual abuse^38^.

### Policy implications

Our findings reveal that suicidal ideation among adolescents in LMICs is a growing global concern, with adolescent girls being particularly vulnerable. This underscores the necessity of implementing initiatives specifically targeting young females^36^. While some interventions have shown improvement, further global efforts are required to reduce suicidal ideation among adolescents. For example, the WHO recommends several strategies to address adolescent suicidal ideation: (1) limiting access to means of suicide; (2) promoting responsible media reporting on suicide; (3) fostering socio-emotional life skills in adolescents; and (4) ensuring early identification, assessment, management, and follow-up for individuals affected by suicidal behaviors. Studies on strategies to prevent suicidal ideation among adolescents have found that awareness and skills training, cognitive behavioral therapy, dialectical behavior therapy, and youth-nominated support teams are effective^39^. Furthermore, it is important to ask adolescents with suicidal ideation about substance use, sleep patterns, specific personality traits, and non-suicidal self-harm. This helps identify those at high risk of attempting suicide and ensures they receive appropriate assistance^40^. Despite the substantial variation among countries, it is urgent to implement national and global policies to reduce adolescent suicidal ideation.

### Limitations and Strengths

While this study provides a thorough overview of global and national trends in adolescent suicidal ideation, the WHO GSHS database is subject to several limitations. First, self-reported questionnaires are prone to reporting bias (e.g., recall bias and social desirability bias), and adolescents may underreport suicidal ideation because of socio-cultural stigma and taboos^41^. Moreover, the degree of underreporting may vary substantially across countries depending on cultural attitudes toward mental health and suicide^42^, as well as the mode of survey (e.g., privacy of the setting, presence of authority figures, and whether the survey was paper-based or electronic-based), further impacting reporting patterns^43^. In addition, suicidal ideation was assessed using a single-item question (“During the past 12 months, did you ever seriously consider attempting suicide?”), which May not fully capture the dynamic, episodic, and personal nature of such thoughts. This Limitation should be considered when interpreting the Magnitude and pattern of change across countries. Second, the analysis is restricted to the 23 countries in which the standard suicidal-ideation item was available and surveyed on at least two occasions between 2003 and 2021; GSHS countries with only one wave or without this item were necessarily excluded, so their temporal patterns could not be assessed. Third, this study is limited to adolescents attending school, and additional research is necessary to understand suicidal ideation among adolescents who are not in school. Lastly, due to the different survey years and durations in each country, caution is necessary when comparing trends between countries. Although these findings provide important insights for future research, it is important to recognize that our analysis may not apply to the current situation. Despite these limitations, the study’s strengths include a large sample size of adolescents from 23 countries across five continents, most of which were nationally representative samples.

## Conclusion

This study provides a global overview of temporal patterns in suicidal ideation among adolescents aged 13 to 15 years across 23 countries. Several countries showed increasing trends in suicidal ideation, with the most notable rises in Myanmar, Guyana, and Saint Vincent and the Grenadines. Differences between boys and girls were also observed in countries such as Argentina, Saint Vincent and the Grenadines, Thailand, Benin, Kuwait, and Guyana. In all of these countries except Guyana, the trend was more unfavorable among girls, as they exhibited either a greater increase or a smaller decrease in suicidal ideation prevalence compared to boys. While this study cannot determine the exact causes of these trends, they may reflect broader social, economic, or political conditions that need further research. These findings emphasize the importance of ongoing monitoring of adolescent mental health. Future research should explore the underlying reasons behind these patterns. Public health efforts that are sensitive to each country’s situation will be important to support the mental well-being of adolescents around the world.

## Supplementary Information

Supplementary Information.

## Data Availability

Data are available on reasonable request. Study protocol and statistical code will be available from DKY (email: [yonkkang@gmail.com](mailto:yonkkang@gmail.com)). Dataset: Available from the World Health Organization and the US Centers for Disease Control and Prevention through a data use agreement. Global School-based Student Health Survey is publicly available as follows link: https://extranet.who.int/ncdsmicrodata/index.php/catalog/gshs/?page=1&ps=15&repo=GSHS.
