# Supplementary material for: Temporal trends and patterns in suicidal ideation among adolescents in 23 countries from 2003 to 2021
Source: Sci Rep. 2025 Oct 8;15:35195. doi: 10.1038/s41598-025-19158-5 (PMC12508072; doi:10.1038/s41598-025-19158-5)
Supplement: Supplementary file 1 — Supplementary Information. [file 41598_2025_19158_MOESM1_ESM.docx]

| **Supplementary Material** |
| --- |

Original Article

**Temporal trends and patterns in suicidal ideation among adolescents in 23 countries from 2003 to 2021**

**Running title:** Suicidal ideation in adolescents

Wonwoo Jang^1,2†^, Yejun Son^1,3†^, Jae E. Lee^1,2^, Hyejun Kim^1,4^, Seohyun Hong^1,2^, Yeona Jo^1,3†^, Hanseul Cho^5^, Hayeon Lee^1,6^, Ho Geol Woo^1,7^, André Hajek^8^, Dong Keon Yon^1,2,3,6,9*^, Lee Smith^10,11*^

^†^ These authors contributed equally to this work as first authors.

* These authors contributed equally to this work as corresponding authors.

*Corresponding authors:

**Dong Keon Yon,** MD, PhD, FACAAI, FAAAAI, ATSF

Department of Pediatrics, Kyung Hee University College of Medicine, 23 Kyungheedae-ro, Dongdaemun-gu, Seoul 02447, South Korea

Email: yonkkang@gmail.com

**Lee Smith,** PhD

Centre for Health, Performance and Wellbeing, Anglia Ruskin University, Cambridge, CB1 1PT, UK

Email: Lee.Smith@aru.ac.uk

**Figure S1**. Residual-versus-fitted plots for linear trend models.


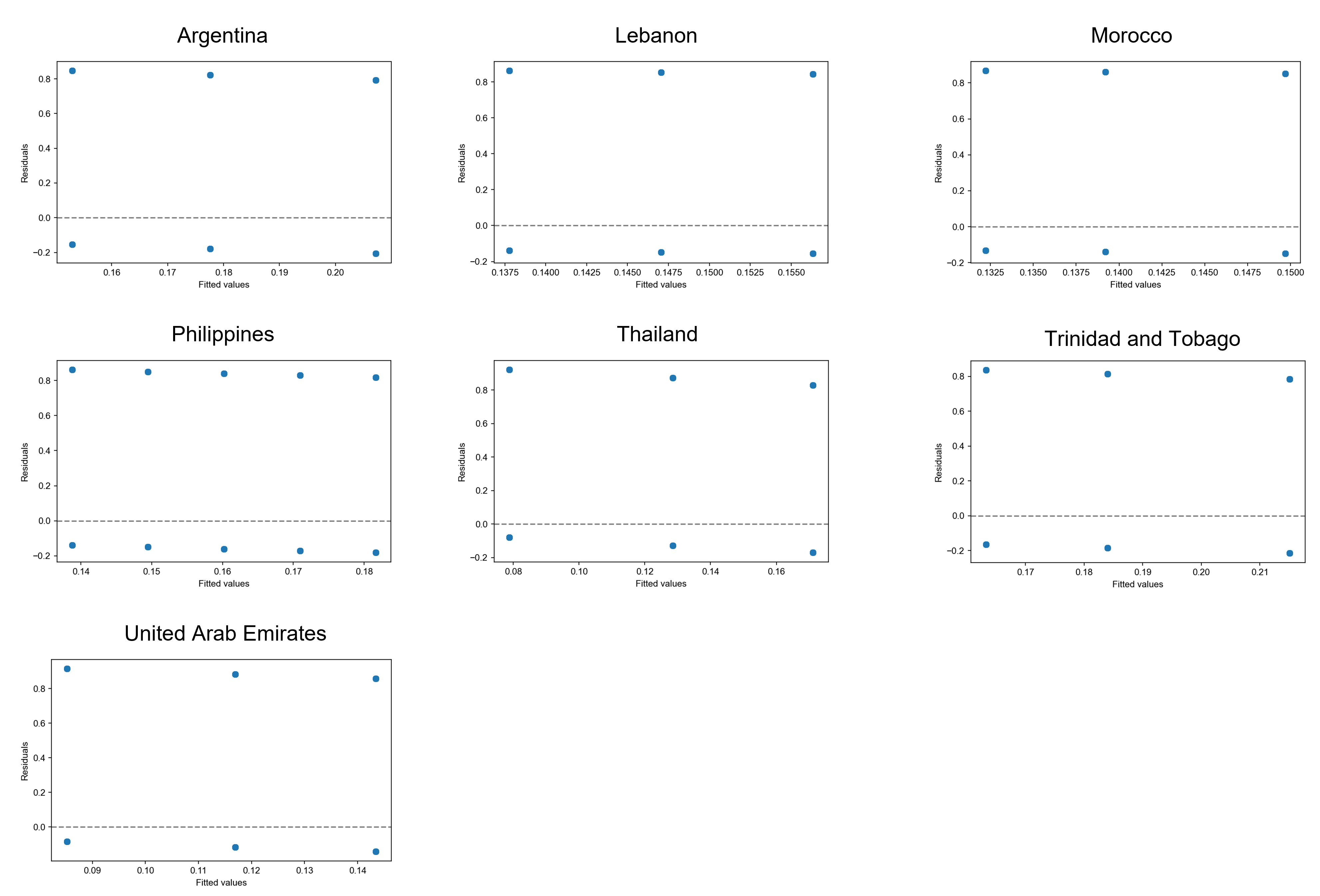


**Table S1**. Linearity diagnostics for countries with ≥ 3 GSHS survey waves

| **Country** | **Waves** | **R² (Lin)** | **AIC (Lin)** | **BIC (Lin)** | **R² (Quad)** | **AIC (Quad)** | **BIC (Quad)** | **p (Quad term)** | **Linearity assessment** |
| --- | --- | --- | --- | --- | --- | --- | --- | --- | --- |
| Argentina | 3 | 0.004 | 10,773.71 | 102,791.67 | 0.004 | 102,752.09 | 102,779.03 | **<0.001** | Non-linear suspected |
| Lebanon | 3 | 0.000 | 7,387.24 | 7,401.27 | 0.000 | 7,389.24 | 7,410.28 | 0.941 | Linear adequate |
| Morocco | 3 | 0.000 | 5,994.49 | 6,008.23 | 0.001 | 5,991.33 | 6,011.94 | **0.023** | Non-linear suspected |
| Philippines | 5 | 0.001 | 25,640.31 | 25,656.42 | 0.006 | 25,545.18 | 25,569.36 | **<0.001** | Non-linear suspected |
| Thailand | 3 | 0.013 | 9,297.33 | 9,311.61 | 0.014 | 9,294.35 | 9,315.78 | **0.026** | Non-linear suspected |
| Trinidad | 3 | 0.003 | 8,983.89 | 8,997.32 | 0.004 | 8,979.08 | 8,999.22 | **0.009** | Non-linear suspected |
| United Arab Emirates | 3 | 0.006 | 14,877.63 | 14,893.00 | 0.011 | 14,785.75 | 14,808.80 | **<0.001** | Non-linear suspected |

*Abbreviation*: AIC, Akaike information criterion; BIC, Bayesian information criterion; GSHS, Global School-based Student Health Survey; p (Quad term), p-value for the quadratic (year²) coefficient in the regression model; R² (Lin), coefficient of determination for the linear model; R² (Quad) coefficient of determination for the quadratic model; Waves, number of survey years available for the country.

^*^ Model fit was assessed with the Akaike and Bayesian information criteria (AIC and BIC); if the quadratic model’s AIC and BIC were at least two points lower than those of the linear model, the quadratic specification was considered the better fit.

^*^ R² (Lin) and R² (Quad) represent the proportion of variance explained by the linear and quadratic models, respectively.

^*^ Bold P-values indicate significant curvature (p < 0.05).
